# Is a higher body mass index associated with longer duration of survival with disability in frail than in non-frail older adults?

**DOI:** 10.1038/s41366-024-01681-6

**Published:** 2024-11-15

**Authors:** Daiki Watanabe, Tsukasa Yoshida, Yuya Watanabe, Yosuke Yamada, Misaka Kimura

**Affiliations:** 1 Faculty of Sport Sciences, Waseda University, 2-579-15 Mikajima, Tokorozawa-city, Saitama 359-1192 Japan; 2 National Institute of Health and Nutrition, National Institutes of Biomedical Innovation, Health and Nutrition, 3-17 Senriokashimmachi, Settsu-city, Osaka 566-0002 Japan; 3 Institute for Active Health, Kyoto University of Advanced Science, 1-1 Nanjo Otani, Sogabe-cho, Kameoka-city, Kyoto 621-8555 Japan; 4 Senior Citizen’s Welfare Section, Kameoka City Government, 8 Nonogami, Yasu-machi, Kameoka-city, Kyoto 621-8501 Japan; 5 National Institute of Biomedical Innovation, National Institutes of Biomedical Innovation, Health and Nutrition, 7-6-8 Saito-Asagi, Ibaraki-city, Osaka 567-0085 Japan; 6 Faculty of Sport Study, Biwako Seikei Sport College, 1204 Kitahira, Otsu-city, Shiga 520-0503 Japan; 7 Laboratory of Applied Health Sciences, Kyoto Prefectural University of Medicine, 465 Kajii-cho, Kamigyo-ku, Kyoto-city, Kyoto 602-8566 Japan

**Keywords:** Geriatrics, Weight management, Epidemiology

## Abstract

**Background/Objectives:**

This study investigated the hitherto unclear association of body mass index (BMI) with age at overall, disability, and disability-free survival in older adults with and without frailty.

**Methods:**

This prospective cohort study enroled 10232 Japanese adults aged ≥65 years, who underwent follow-up for adverse events, from the Kyoto-Kameoka Study conducted from 2011–2016. BMI, calculated based on self-reported height and body weight, was classified into five categories: <18.5, 18.5–21.4, 21.5–24.9, 25.0–27.4, and ≥27.5 kg/m^2^. Frailty was assessed using the validated Kihon Checklist. The relationships between BMI and disability and mortality were analysed using multivariate Cox proportional hazards models and Laplace regression.

**Results:**

During the 5.3-year median follow-up period (45472 person-years), 2348 (22.9%) incidences of disabilities occurred. After adjusting for confounders, including medical history and lifestyle, individuals in the lowest and highest BMI categories had a higher hazard ratio (HR) of disability [<18.5 kg/m^2^: HR: 1.31, confidence interval (CI): 1.16–1.49; ≥27.5 kg/m^2^: HR: 1.27, 95% CI: 1.08–1.49, *p* for non-linearity <0.001] compared with that of those with BMI = 21.5–24.9 kg/m^2^. In the 50th percentile differences in age at overall and disability-free survival, participants with BMI < 18.5 kg/m^2^ were more likely to die before disability incidence [survival with disability (overall survival – disability-free survival): −10.2 months]; those with BMI ≥ 27.5 kg/m^2^ had longer survival with disability (12.5 months). These relationships were more marked in the frailty-stratified model, where in the BMI ≥ 27.5 kg/m^2^ group, individuals with frailty survived longer with disability (27.2 months) than did individuals without frailty (6.2 months).

**Conclusion:**

Higher BMI is associated with a longer duration of survival with disability among older adults, especially in those with frailty. Therefore, reversing frailty should be prioritised because individuals with frailty have a shorter probability of disability-free survival than do individuals without frailty, regardless of BMI.

## Introduction

Frailty, characterised by the loss of integrity and impaired function of multiple physiological systems [1–3], is a geriatric syndrome closely associated with mortality [4–7]. Its prevalence increases with age [4, 5] and is 12–24% in individuals aged ≥50 years [8]. Thus, accumulating evidence on appropriate care for preserving functional independence is required for prolonging lifespan for older adults with frailty [9].

Body mass index (BMI) is a convenient measure for evaluating thinness and fatness. The mean BMI differs among geographical regions, such as America, Europe, and Japan [10]. Furthermore, compared with the White and Black population, Asian population shows onset of type 2 diabetes at a lower BMI [11], thus suggesting that the cut-off values of BMI for predicting obesity-related adverse events vary geographically. Hence, extrapolating the results of previous studies conducted in non-Asians to Asians, including Japanese, becomes difficult [12].

Previous studies have reported inconsistent results on the association between BMI and the risk of disability in older adults, irrespective of the geographical region [13–17]. Higher BMI is associated with higher frailty levels [5, 18], while frailty also bears a strong association with disability [19]. Reportedly, overweight and obesity are inversely associated with mortality [20, 21] and falls [22] in individuals with frailty. Thus, some excess body weight is associated with longevity among older adults, especially in individuals with frailty. However, to the best of our knowledge, differences in the association of BMI with the age at overall, disability, and disability-free survival between older adults with and without frailty have not been thoroughly examined.

Our study objectives were (1) to evaluate the dose–response association between BMI and disability risk, and (2) to investigate the relationships between BMI and age at overall, disability, and disability-free survival in older adults with and without frailty. We hypothesised that both higher and lower BMI ranges would be associated with disability. Therefore, because BMI is associated with longevity [20, 21], less years with disability-free survival [14], we speculated that older adults with frailty with a higher BMI would have longer survival with disability than that of those with other BMI ranges.

## Materials and methods

### Study population and assessment of baseline characteristics

**Fig. 1:**
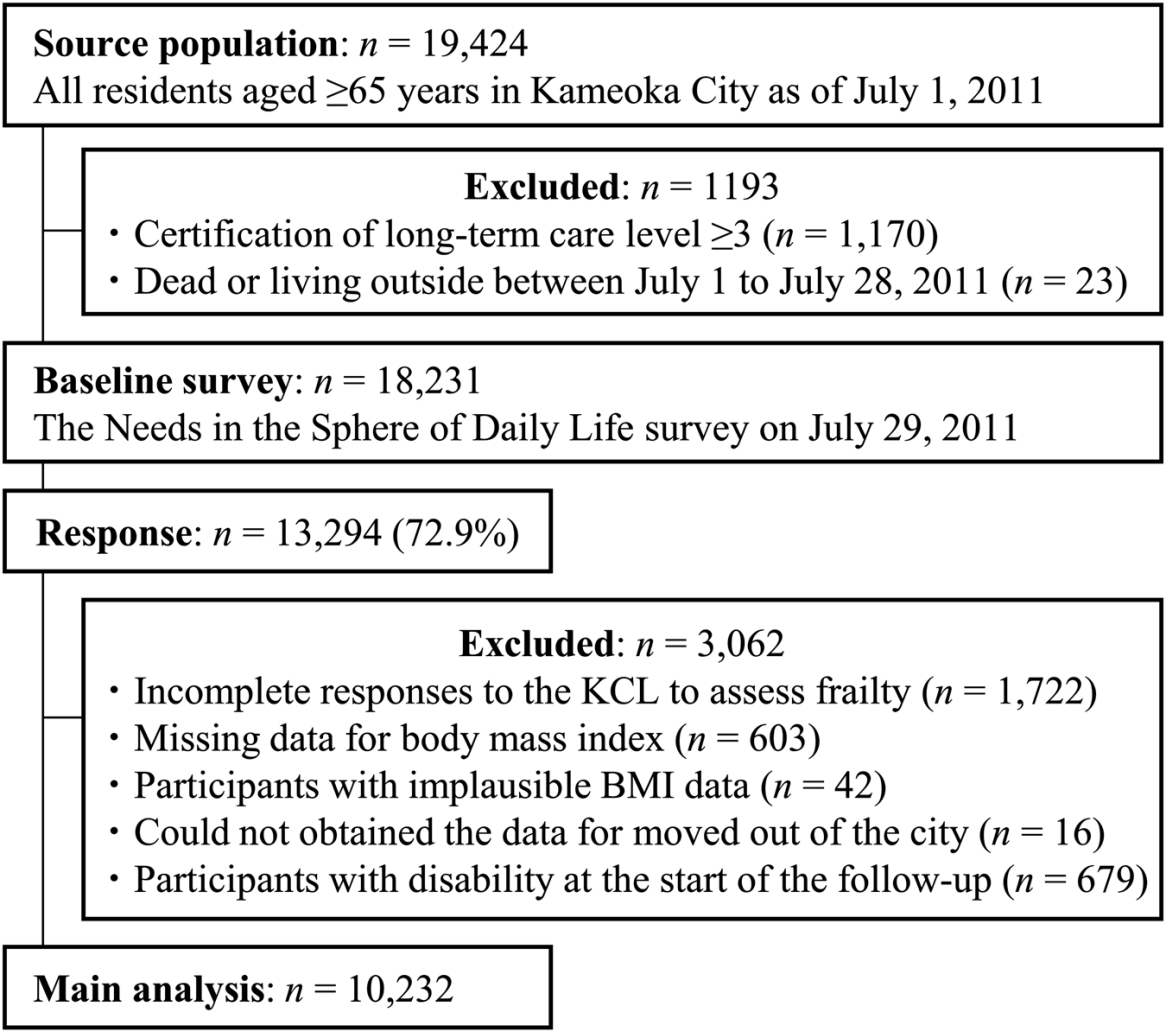
Participant flow diagram for the analysis of the relationship between body mass index and disability in the Kyoto-Kameoka study. BMI body mass index, KCL Kihon checklist.

The Kyoto-Kameoka study is a population-based, prospective cohort study of individuals aged ≥65 years, residing in Kameoka City, Kyoto Prefecture, Japan. The details of this cohort study have been reported previously [6, 18, 21, 23–26]. Briefly, to conduct an appraisal of all residents of Kameoka City aged ≥65 years on 1 July 2011, a municipal employee in charge of the survey selected qualified candidates based on the individual’s name, sex, and date of birth; this information was obtained from the Basic Resident Register maintained by Kameoka City Hall (Fig. 1). The Needs in the Sphere of Daily Life survey (baseline survey), which includes the self-reported height, body weight, and frailty assessment tools [Kihon Checklist (KCL)], was mailed to the residents of Kameoka on 29 July 2011. Of these potential candidates (*n* = 18,231), 13,294 responded to the survey (response rate: 72.9%) by mail. We excluded individuals with incomplete responses to the KCL (*n* = 1,722), missing BMI data (*n* = 603), those who reported implausible BMI data [BMI < 14 or ≥40 kg/m^2^; *n* = 42) [18, 21], whose data could not obtained owing to relocation from the city (*n* = 16), and those who had disability at the commencement of follow-up (n = 679). Finally, 10,232 participants (5459 women; 4773 men) were included in the study.

This study was conducted in accordance with the Declaration of Helsinki and was approved by the research ethics committees of the National Institutes of Biomedical Innovation, Health, and Nutrition (NIBIOHN-76-2), Kyoto University of Advanced Science (20-1), and Kyoto Prefectural University of Medicine (RBMR-E-363). Informed consent were received by mail from each participant along with the completed questionnaires.

### Evaluation of BMI

BMI was calculated by dividing the self-reported body weight by the height squared (kg/m^2^). Previously, we found no significant difference between the BMI calculated from self-reported height and body weight and the objectively measured values in a sub-cohort of the Kyoto-Kameoka Study through clustered random sampling (mean difference: 0.5 kg/m^2^ in women and 0.4 kg/m^2^ in men; Pearson’s rank correlation coefficient between the BMI values: 0.912 for women, 0.916 for men) [18]. The interclass correlation coefficients, employed as a reproducibility scale of the self-reported BMI obtained from the baseline and additional surveys, were 0.888 and 0.910 for women and men, respectively [18]. For the Bland-Altman plot of agreement between self-reported and measured BMI, there was no variation in agreement in both sexes based on the BMI magnitude (Supplementary Fig. 1). We classified participants into the following BMI categories according to a previous study [21]: <18.5, 18.5–21.4, 21.5–24.9, 25.0–27.4, and ≥27.5 kg/m^2^. This is because if using the international BMI cut-off value for obesity (≥BMI 30 kg/m^2^) in our older Japanese population [27], only few would be considered as individuals with obesity (181 out of 10,232 [1.8%]). Furthermore, people with a ≥BMI 25 kg/m^2^ are defined as having obesity in Japan [27].

### Assessment of frailty status

Frailty was defined as a score ≥7 on the 25-item, validated self-administered KCL [24, 28]. The KCL, based on a deficit accumulation model [6], assesses frailty from a multidimensional perspective, including cognitive, social, and depressive factors, in addition to physical aspects. The KCL encompasses seven subdomains: instrumental activities of daily living disability, malnutrition, physical inactivity, oral dysfunction, cognitive domain, socialisation domain, and depression. Difficulty in each activity or function is assigned one point, with the KCL score varying from 0 (no frailty) to 25 (high frailty). We have previously confirmed the good predictive ability of the KCL for frailty, defined by the revised Japanese version of the Cardiovascular Health Study criteria according to the Fried phenotype model in a sub cohort of the Kyoto-Kameoka study, that measured grip strength and gait speed (area under the receiver operating characteristic curve = 0.861; sensitivity = 76.2%; specificity = 79.9%) [24]. A prospective study demonstrated a dose response-dependent, strong log-linear relationship between the KCL score and mortality [6].

### Disability and mortality status

Disability incidence was identified using the long-term care insurance system’s nationally unified database in Japan [29]. This system defines disability ( ≥ support level 1) as a condition in which some assistance is required for performing instrumental daily living activities. Local government officials performed in-person assessment of daily functioning in candidate individuals with disability using a 74-item questionnaire, based on the activities of daily living. Based on the questionnaire results and the physician’s opinion, the candidates’ functional disability level was determined by the Long-Term Care Insurance Certification Committee, comprising of academic experts in healthcare and welfare. This disability level information was provided by Kameoka City Hall officials. The survival status was assessed using data from the Basic Resident Register maintained by the Kameoka City Hall, collected between 30 July 2011 and 30 November 2016. Residents whose records were removed for administrative purposes or those who had moved out of the municipality were censored.

### Other covariates

All covariates were obtained from questionnaire data from a baseline survey [26]. We collected data on the following basic characteristics: smoking status (“Do you smoke?”: almost daily; sometimes; used to, but quit; never); drinking status (“Do you drink alcohol?”: almost daily, sometimes, almost never, never); sleep duration (minutes); living status (“What is your family structure?”: living alone, living with family, other); education attainment (years); socioeconomic status (“Financially, how does your life feel currently?”: hard, somewhat hard, somewhat easy, easy); oral status (“Do you use dentures?”: yes, no); taking medication (number); and chronic disease [“Do you have a disease (presence of hypertension, stroke, heart disease, diabetes, hyperlipidaemia, gastrointestinal disease, respiratory disease, urological diseases, and cancer]?”: yes, no). Comorbidity scores were calculated from the data obtained on the nine comorbidity statuses. The summed value yielded a total score ranging from 0 (no comorbidity) to 9 (poor status). The previous week’s physical activity (PA) and sitting time per day were evaluated using the International Physical Activity Questionnaire-Short Form.

### Statistical analysis

The participants’ descriptive statistics for continuous and categorical variables were presented as the mean with standard deviation and frequency with proportion, respectively. Missing indicators were created if information on covariates pertaining to smoking status (*n* = 195; 1.9%), alcohol consumption (*n* = 161; 1.6%), PA (*n* = 228; 2.2%), sitting time (*n* = 1075; 10.5%), sleep time (*n* = 484; 4.7%), family structure (*n* = 709; 6.9%), educational attainment (*n* = 1034; 10.1%), socioeconomic status (*n* = 369; 3.6%), denture use (*n* = 111; 1.1%), and medications (*n* = 558; 5.5%) was missing.

We calculated each participant’s person-years of follow-up from the date on which BMI was obtained to the date of disability and death, relocation from the study area, or end of follow-up, whichever occurred first. The rate of disability and all-cause mortality for each BMI group was presented as the number of events per 1000 person-years. We employed a multivariate Cox proportional hazards model that included baseline covariates to adjust for confounders associated with BMI and mortality. The assumptions of the Cox proportional hazards model were confirmed using the Schoenfeld residual test (*p* = 0.255). The results of these analyses were presented as hazard ratios (HRs) with 95% confidence intervals (CIs), calculated using the 21.5–24.9 kg/m^2^ BMI group as reference, based on previous studies [18, 21, 30]. The *p*-value of the linear trend was calculated by considering BMI exposure as a continuous variable. Furthermore, to evaluate the curvilinear relationship between BMI and disability, we used a restricted cubic spline model with three knots (10th, 50th, and 90th percentiles) based on the distribution of baseline BMI. These results were presented as HRs (95% CI), with the HR calculated using BMI = 23.0 kg/m^2^ as reference [21]. The statistical significance of nonlinearity was assessed using Wald’s test to compare the likelihood ratio of the spline model with the linear model, and *p* < 0.05 indicated a statistically significant non-linear relationship between the exposure and outcome [21, 25].

Furthermore, 50th percentile differences (PDs) in age at disability and death were calculated according to the BMI groups using the Laplace regression model. The PDs represented differences in the period until the first 50% of events occurred. This analysis adjusted covariates for model 2 using the ‘laplacereg’ command in STATA [31]. Median PDs (months) for overall and disability-free survival in each BMI group were calculated using the 21.5–24.9 kg/m^2^ group as reference. To calculate the duration of survival with disability, we calculated the difference of the 50th PDs of disability and death events using the following equation: 50th PD of overall survival—50th PD of disability-free survival. If the results were greater than 0 (value is +), we inferred that the duration of survival with disability was longer; if lower than 0, participants were more likely to die before disability incidence.

We performed sensitivity analysis using the following three methods: (1) to eliminate the possibility of reverse causal relationships, we excluded disability events (406 men and 712 women) recorded in the first 2 years of follow-up, (2) we performed a similar analysis using a dataset wherein missing values for covariates were replaced with multiple imputations, and (3) we used the multivariable sub-distribution hazard model approach proposed by Fine and Gray as the competing risk model. Multiple imputation analysis comprised the results of pooled analyses of 20 datasets, created with random numbers using the multiple-imputation method to replace the missing values of covariates using the ‘mi estimate’ command in STATA. All missing values were presumed to be missing at random. As mortality events might compete with these relationships when assessing the relationship between the exposure variables and the incidence of disability, disability was set as the event of interest and mortality as a competing event in competing risk model.

Multivariate analysis, which averts multicollinearity, was conducted by modelling potential confounders reported previously [20–22]. Model 1 was adjusted for age (continuous), sex (women or men), and population density ( ≥ 1000 or <1000 people/km^2^). Model 2 was adjusted for all variables from model 1 plus smoking status (never-smoker, past-smoker, current-smoker, missing), alcohol consumption (never-drinker, almost never-drinker, current-drinker, missing), PA ( < 150, 150–299, ≥300 min/week, missing), sitting time ( < 5, 5 to <7, 7 to <9, ≥9 h/d, missing), sleep duration (<360, 360 to <420, 420 to <480, ≥480 min/d, missing), family structure (living alone, living with others, missing), education level ( ≤ 9, 10–12, ≥13 years, missing), economic status (high, low, missing), denture use (yes, no, missing), medication use (none, 1, 2, 3, 4, ≥5, missing), number of chronic diseases (continuous), and frailty status (yes or no).

Statistical analyses were performed using STATA MP, Version 15.0 (StataCorp LP, College Station, TX, USA), and a two-tailed probability of <5% was considered significant.

## Results

Table 1 presents the participants’ characteristics according to the BMI categories. Participants with BMI ≥ 27.5 kg/m^2^ were younger, and the proportion of current smokers, denture users, medication non-users, and high economic status was lower than in individuals with BMI < 18.5 kg/m^2^. The prevalence of frailty in the BMI < 18.5, 18.5–21.4, 21.5–24.9, 25.0–27.4, and ≥27.5 kg/m^2^ groups was 61.6%, 40.6%, 34.7%, 36.9% and 51.0%, respectively; thus, the prevalence of frailty was high in individuals with high and low BMI. These results were similar to those of the prevalence of subdomains, such as physical, cognitive, and depression, included in the KCL (Supplementary Table 1). Participants excluded from the analysis were older and were predominantly women compared to those who were included (Supplementary Table 2).

**Table 1:** Baseline characteristics of the study participants according to body mass index.

	Total (*n* = 10,232)		BMI categories (kg/m^2^)									
			<18.5 (*n* = 933)		18.5–21.4 (*n* = 2882)		21.5–24.9 (*n* = 4370)		25.0–27.4 (*n* = 1413)		≥27.5 (*n* = 634)	
Age [years]^a^	73.6	(6.5)	76.3	(7.5)	74.1	(6.9)	73.0	(6.0)	73.0	(6.0)	72.7	(5.9)
Women [*n* (%)]^b^	5459	(53.4)	638	(68.4)	1668	(57.9)	2124	(48.6)	660	(46.7)	369	(58.2)
PD ≥ 1000 people/km^2^ [*n* (%)]^b^	4616	(45.1)	413	(44.3)	1270	(44.1)	2026	(46.4)	622	(44.0)	285	(45.0)
Height [cm]^a^	156.9	(9.0)	154.3	(8.6)	156.4	(9.0)	157.8	(8.8)	157.8	(9.0)	155.7	(10.0)
Body weight [kg]^a^	55.7	(10.3)	41.2	(5.3)	49.4	(6.1)	57.6	(6.9)	64.9	(7.5)	71.7	(9.9)
Body mass index [kg/m^2^]^a^	22.5	(3.2)	17.2	(1.0)	20.1	(0.8)	23.1	(1.0)	26.0	(0.7)	29.5	(2.0)
Current smoker [*n* (%)]^b^	1169	(11.4)	117	(12.5)	357	(12.4)	488	(11.2)	147	(10.4)	60	(9.5)
Current alcohol drinker [*n* (%)]^b^	3960	(38.7)	257	(27.6)	1022	(35.5)	1851	(42.4)	612	(43.3)	218	(34.4)
Physical activity [min/week]^a^	216	(419)	129	(308)	206	(395)	248	(454)	222	(427)	160	(371)
Sitting time [min/day]^a^	327	(229)	365	(255)	323	(234)	318	(220)	318	(215)	373	(243)
Sleep time [min/day]^a^	408	(89)	416	(107)	410	(88)	406	(85)	405	(87)	400	(90)
Living alone [*n* (%)]^b^	1161	(11.4)	107	(11.5)	363	(12.6)	472	(10.8)	146	(10.3)	73	(11.5)
Education ≥13 y [*n* (%)]^b^	2076	(20.3)	143	(15.3)	576	(20.0)	941	(21.5)	289	(20.5)	127	(20.0)
HSES [*n* (%)]^b^	3254	(31.8)	317	(34.0)	936	(32.5)	1394	(32.0)	438	(31.0)	169	(26.7)
Denture use [*n* (%)]^b^	6095	(59.6)	612	(65.6)	1753	(60.8)	2559	(58.6)	820	(58.0)	351	(55.4)
No medication [*n* (%)]^b^	2134	(20.9)	196	(21.0)	701	(24.3)	913	(20.9)	243	(17.2)	81	(12.8)
Hypertension [*n* (%)]^b^	3920	(38.3)	224	(24.0)	876	(30.4)	1767	(40.4)	711	(50.3)	342	(53.9)
Stroke [*n* (%)]^b^	429	(4.2)	36	(3.9)	110	(3.8)	195	(4.5)	60	(4.3)	28	(4.4)
Heart disease [*n* (%)]^b^	1261	(12.3)	105	(11.3)	311	(10.8)	532	(12.2)	200	(14.2)	113	(17.8)
Diabetes [*n* (%)]^b^	1088	(10.6)	69	(7.4)	244	(8.5)	470	(10.8)	178	(12.6)	127	(20.0)
Hyperlipidaemia [*n* (%)]^b^	970	(9.5)	52	(5.6)	244	(8.5)	438	(10.0)	148	(10.5)	88	(13.9)
Respiratory disease [*n* (%)]^b^	482	(4.7)	68	(7.3)	133	(4.6)	187	(4.3)	64	(4.5)	30	(4.7)
Digestive disease [*n* (%)]^b^	850	(8.3)	133	(14.3)	267	(9.3)	330	(7.6)	85	(6.0)	35	(5.5)
Urological diseases [*n* (%)]^b^	640	(6.3)	66	(7.1)	176	(6.1)	272	(6.2)	88	(6.2)	38	(6.0)
Cancer [*n* (%)]^b^	387	(3.8)	58	(6.2)	127	(4.4)	138	(3.2)	46	(3.3)	18	(2.8)
No. of chronic diseases^a,c^	0.98	(0.99)	0.87	(0.99)	0.86	(0.93)	0.99	(1.00)	1.12	(1.01)	1.29	(1.11)
Frailty [n (%)]^b^	4105	(40.1)	575	(61.6)	1170	(40.6)	1516	(34.7)	521	(36.9)	323	(51.0)

The number of missing values for variables is as follows: smoking status (*n* = 195; 1.9%), alcohol drinker (*n* = 161; 1.6%), physical activity (*n* = 228; 2.2%), sitting time (*n* = 1075; 10.5%), sleep time (*n* = 484; 4.7%), family structure (*n* = 709; 6.9%), educational attainment (*n* = 1034; 10.1%), socioeconomic status (*n* = 369; 3.6%), denture use (*n* = 111; 1.1%), and medications (*n* = 558; 5.5%). Body mass index was calculated as body weight (kg) divided by height squared (m^2^).

*PD* population density, *HSES* high socioeconomic status.

^a^Continuous values are presented as the mean (standard deviation).

^b^Categorical values are presented as numbers (percentages).

^c^The comorbidity scores were summed to obtain a total score ranging from 0 (no comorbidity) to 9 (poor status) from the data obtained on disease status (including the presence of hypertension, stroke, heart disease, diabetes, hyperlipidaemia, digestive disease, respiratory disease, urological diseases, and cancer).

**Fig. 2:**
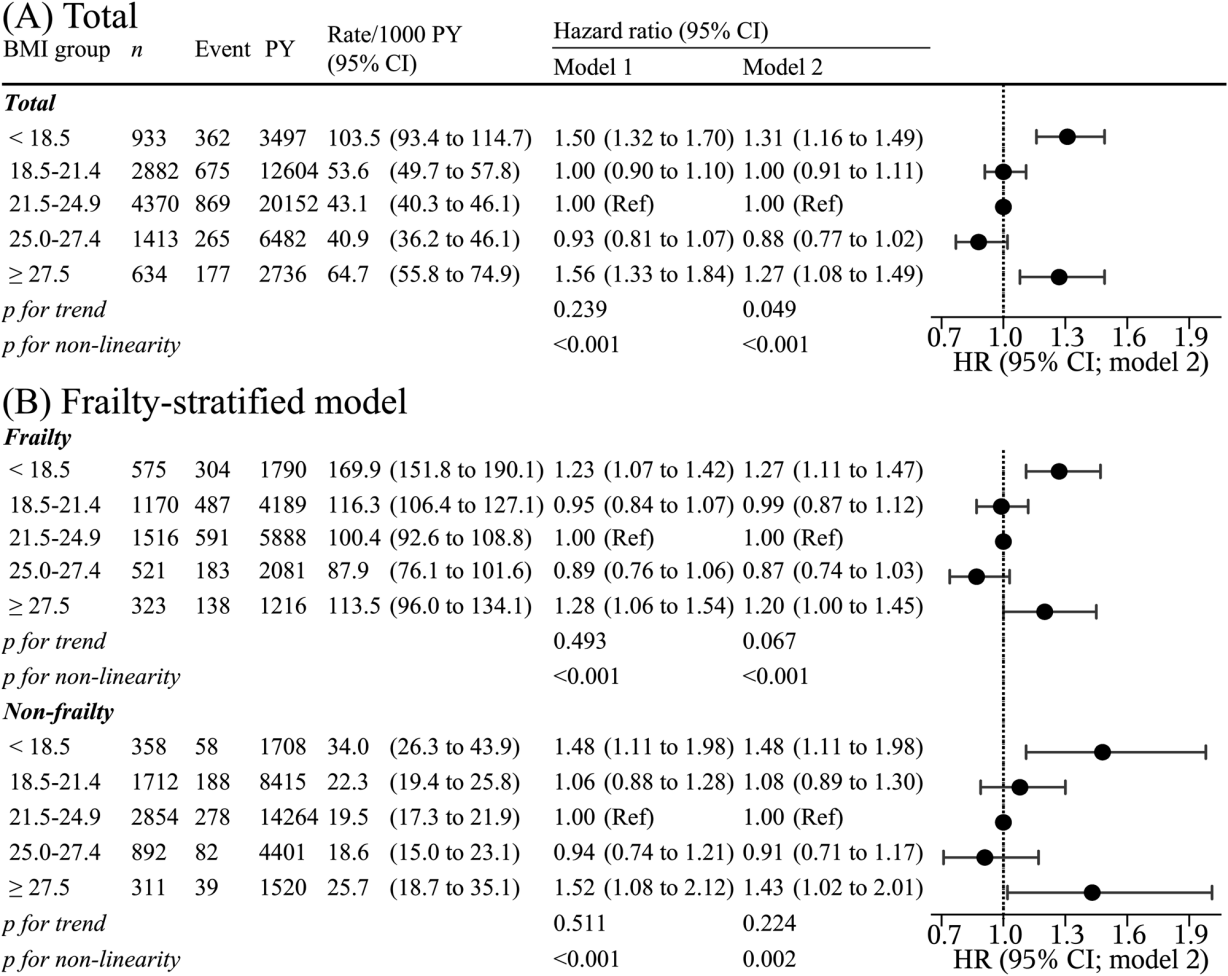
Hazard ratios for disability calculated using multivariable Cox proportional hazards analysis based on body mass index. **A** total participants (**B**) frailty stratified model. Model 1 was adjusted for age, sex, and population density; Model 2 was additionally adjusted for smoking status, alcohol consumption, physical activity, sitting time, sleep duration, family structure, educational level, economic status, denture use, medication use, chronic disease count and/or frailty status. The X-axis of the plot is on a log scale. BMI body mass index, CI confidence interval, HR hazard ratio, PY person-years, Ref reference.

**Fig. 3:**
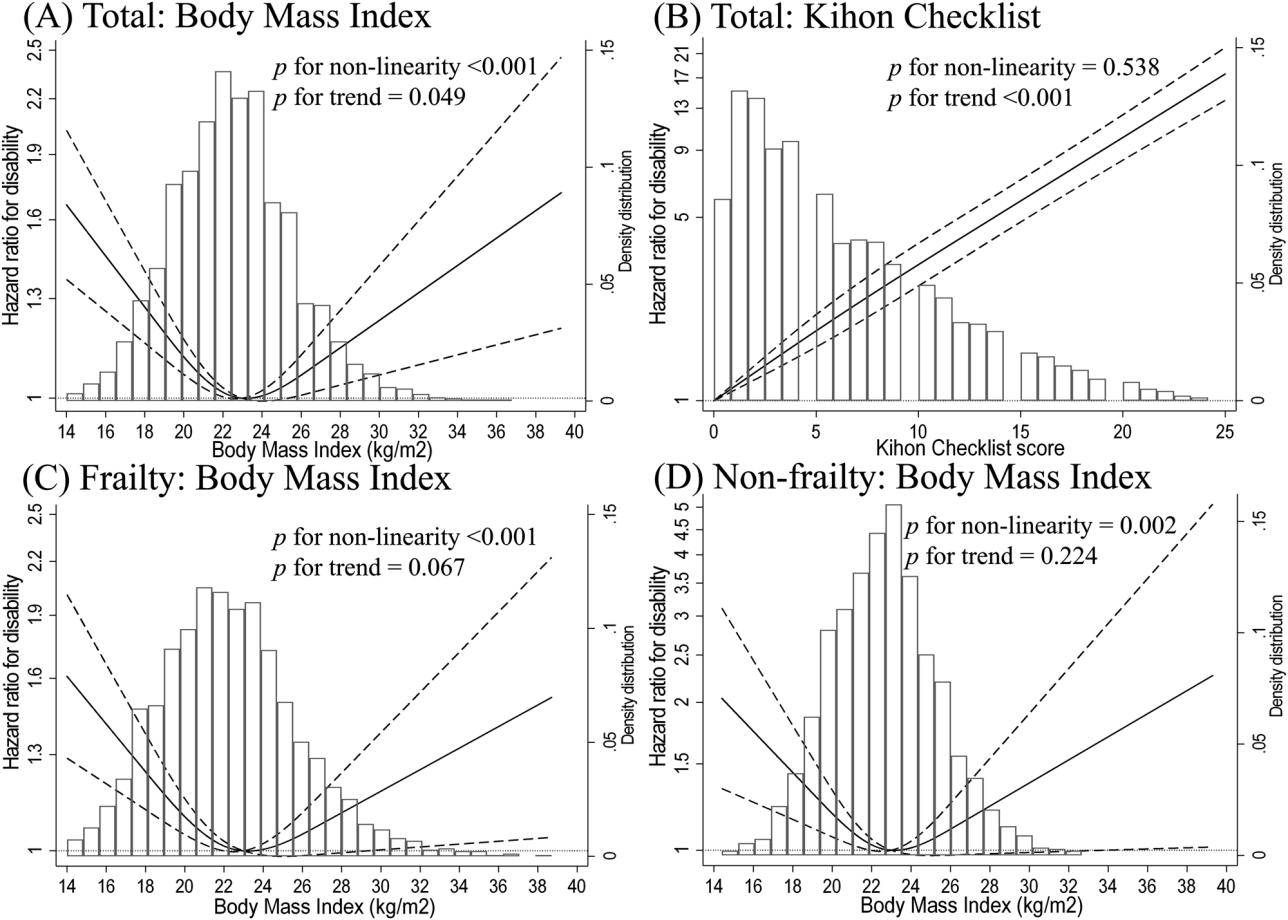
Multivariable adjusted restricted cubic spline model for body mass index and frailty level and disability risk among older adults. Body mass index and disability risk among (**A**) total participants, individuals with (**C**) frailty and (**D**) non-frailty. **B** frailty level and disability risk in total participants. The histogram shows the distribution of body mass index or Kihon checklist score. Solid lines represent hazard ratios, and dashed lines represent 95% confidence intervals (CIs). The hazard ratio was calculated based on a body mass index of 23.0 kg/m^2^ and Kihon checklist score of 0 point as reference. The adjustment factors are age, sex, population density, smoking status, alcohol consumption status, physical activity, sitting time, sleep time, family structure, educational attainment, economic status, denture use, medication use, number of chronic diseases, and/or frailty status.

The relationships between BMI and disability are depicted in Figs. 2A, 3A. The median follow-up period was 5.3 years (interquartile range: 4.2–5.3 years). The total follow-up period was 45,472 person-years; 2348 (22.9%) incident disabilities occurred during the study period. After adjusting for confounders, such as medical history and lifestyle, individuals in the lower and higher BMI groups had a higher HR for disability than did those in the BMI = 21.5–24.9 kg/m^2^ group. Similar results were achieved using the restricted cubic spline model (Fig. 3A). The spline analysis model fit the data better than did the linear regression analysis (Akaike information criterion: 39,268 vs 39,287). A strong dose–response-dependent, log linear relationship was observed between KCL scores and disability risk (Fig. 3B), and individuals with frailty had a higher HR for disability than did those without (HR: 2.42, 95% CI: 2.18–2.68).

Figures 2B and 3C, D depict the relationship between BMI and disability in individuals with and without frailty. Higher and lower BMI were associated with the risk for disability in such individuals. Similar results were obtained in the sensitivity analyses (Supplementary Tables 3–5). In the spline model, older adults with and without frailty exhibited the lowest HR for disability within a BMI range of 22.5–23.5 kg/m^2^ (Fig. 3C, D).

**Fig. 4:**
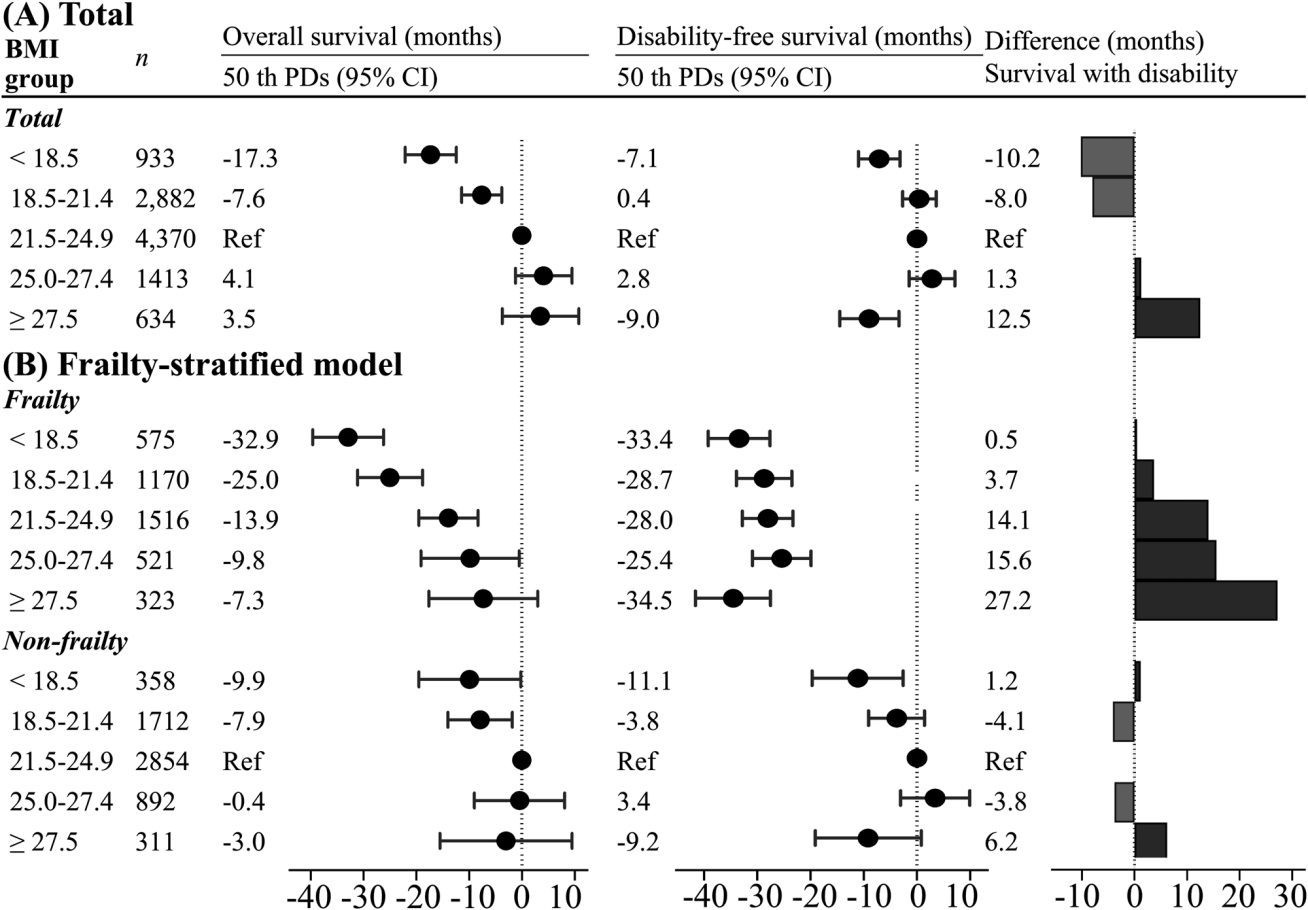
Forest plot and bar chart of the 50th percentile differences in age at disability and death for frailty and body mass index (BMI) status, calculated using the multivariable Laplace regression model. **A** total participants (**B**) frailty stratified model. Results are presented as percentile differences (PDs) (95% confidence interval [CI]). To calculate the duration of survival with disability, we calculated the difference in the 50th PDs of disability and death events using the following equation: 50th PD of overall survival—50th PD of disability-free survival. If the results are greater than 0 (value is +), the duration of survival with disability is inferred to be longer. If the value is lower than 0, the participant is more likely to die before disability incidence. The adjustment factors are age, sex, population density, smoking status, alcohol consumption status, physical activity, sitting time, sleep time, family structure, educational attainment, economic status, denture use, medication use, number of chronic diseases, and/or frailty status.

The relationships between BMI and 50th PDs in age at disability and death are shown in Fig. 4A, B and Supplementary Table 6. For the overall study population, the 50th PDs (95% CIs) in age at overall survival were 0 month (reference) for BMI = 21.5–24.9 kg/m^2^, −17.3 (−22.1 – −12.4) months for BMI < 18.5 kg/m^2^, and −7.6 (–11.4 to –3.8) months for BMI = 18.5–21.4 kg/m^2^ (Fig. 4A). Compared to BMI = 21.5–24.9 kg/m^2^, age at disability free-survival was –7.1 (–11.0 to –3.2) months shorter for BMI < 18.5 kg/m^2^ and –9.0 (–14.5 to −3.4) months shorter for BMI ≥ 27.5 kg/m^2^. Participants with BMI < 18.5 kg/m^2^ were more likely to die before disability incidence (survival with disability: –10.2 months) and those with BMI ≥ 27.5 kg/m^2^ had longer survival with disability (12.5 months). In the frailty status-stratified analyses, even a frail individual with optimal BMI faced a shorter of disability-free survival than did non-frail individuals with higher and lower BMI, respectively (Fig. 4B). In addition, individuals with frailty (27.2 months) with higher BMI had longer survival with disability than did those without (6.2 months).

## Discussion

This study demonstrated that higher and lower BMI was associated with disability: older adults with BMI ≥ 27.5 kg/m^2^ had longer survival with disability, but those with BMI < 18.5 kg/m^2^ were more likely to die before disability incidence. Moreover, individuals with frailty and higher BMI had longer survival with disability than did those without frailty. To the best of our knowledge, this is the first study to demonstrate the existence of a dose–response relationship between BMI and disability in older individuals and that the duration of overall, disability, and disability-free survival differs with respect to BMI and frailty status.

We found that the HR for disability was the lowest at a BMI of 22.5–23.5 kg/m^2^ in older adults with and without frailty. Although results are inconsistent on the relationship between BMI and disability [13–17], a meta-analysis showed that BMI within the range of 23.0–28.0 kg/m^2^ was associated with the lowest HR for death and disability [32]. Given that Asians have a lower BMI optimal for disease prevention than do non-Asians [11], this finding approximates our results. Additionally, our findings indicated that participants with BMI < 18.5 kg/m^2^ were more likely to die before disability incidence and those with BMI ≥ 27.5 kg/m^2^ had longer survival with disability, especially those with frailty. Our previous [21] and other Japanese cohort studies [30] showed that BMI in the range of 23.0–24.0 kg/m^2^ and 22.0–24.9 kg/m^2^ is associated with the lowest mortality risk in older adults. Similar results were reported by two meta-analyses [33, 34]. Our previous [21] and other studies [20] showed that a higher BMI is inversely associated with mortality in individuals with frailty but not in those without, thus supporting our results and suggesting that higher BMI is associated with longer survival with disability among older adults, especially in individuals with frailty. Therefore, based on the current and previous studies [21, 30, 32, 33, 35], the optimal BMI range associated with a healthy life expectancy is 22.5–24.0 kg/m^2^, irrespective of the frailty status in older adults.

Although the mechanisms underlying the U-shaped relationships between BMI and disability are unclear, based on the results of previous studies, two factors associated with energy balance may be held accountable. BMI reflects the dynamic equilibrium of energy balance between energy intake and total energy expenditure [36]. First, a lower BMI may reflect lower energy intake because energy intake lower than the total energy expenditure leads to weight loss. Energy intake must be commensurate with energy requirements to maintain nutritional status and skeletal muscle mass in older adults [37]. Individuals with lower BMI cannot fully store skeletal muscle mass and fat [38], resulting in inadequate energy reserves available for mobilisation in acute stress events. Skeletal muscle mass is inversely associated with disability risk [39], possibly contributing to disability risk because of a lack of energy.

Second, a higher BMI may reflect lower energy expenditure or higher energy intake. PA accounts for approximately 20–35% of the total energy expenditure [40], and people with obesity and overweight are more likely to engage in a lower amount of PA. Objectively measured PA bears an inverse association with disability in older adults [41, 42]. An intervention study showed that the disability prevention effect of PA is more pronounced in older adults with poor lower limb function [43]. High energy intake [23] and obesity [18] are associated with frailty. The Wisconsin Primate Calorie Restriction study indicated that calorie restriction prevented a decline in PA and reduced the incidence of frailty compared to that with ad libitum feeding in rhesus monkeys [44], suggesting that high energy intake and obesity may accelerate ageing, thus supporting our results.

Our study strength was that it evaluated the relationship between BMI and disability in older individuals aged ≥65 years from Kameoka city’s large-scale population-based cohort, using validated self-reported BMI [18] and frailty assessment tools [24]. This approach probably minimised misclassification of BMI groups and frailty status due to self-report bias. However, the study contained certain methodological limitations. First, baseline survey data could not be collected from all residents who were sent the questionnaire. We observed differences in individual characteristics, such as age and sex, between residents who were excluded due to missing exposure or outcome variables and those who were included in the study. This suggests that our participants were more health-conscious than were older people in the general population. Second, BMI evaluation was performed only at baseline, and the participants’ BMI might have changed during the follow-up period, leading to misclassification of BMI and potentially attenuating the relationship between BMI and disability in the exposure assessment. Despite this limitation, this study affirmed the relationship between BMI and disability in both the main and sensitivity analyses. Third, the follow-up period was relatively short; this may result in overestimation of the relationships between the exposure variables and outcomes and inversion of the causal relationship [45] because the HR estimated from the analysis may change with time. However, we confirmed proportional hazards for the relationship between BMI and disability, and the sensitivity analysis that excluded disability events occurring after first 2 years of follow-up showed similar results. Finally, although our study adjusted for several confounders, residual confounders may persist in the association between steps and disability.

Although some epidemiological studies have shown a U-shaped relationship between BMI and all-cause mortality in adults [30, 33, 35, 46], the optimal BMI associated with the lowest risk of mortality increased with age in both Japanese [30, 46] and other populations [33]. Reportedly, a higher BMI may confer protection against the risk of mortality in older individuals with frailty than in those without [20, 21], thus supporting the obesity paradox in older adults with frailty [47]. However, it has been also argued that the obesity paradox is induced by the following two biases [48]: 1) the influence of differences in individuals characteristics, such as people with obesity being younger and more well-nourished than are people without obesity, and 2) the influence of collider stratification bias, which leads to a spurious association between exposure factors and outcomes by stratifying by a third variable, that is associated with both the exposure variable (BMI) and the confounding factor and is downstream from both. Additionally, individuals who are constantly overweight or are undergoing constant weight loss have a higher mortality risk compared to that of those with a constant normal weight, thereby suggesting that the obesity paradox may not be observed when weight trajectories are considered [49]. Therefore, when evaluating the relationship between BMI and mortality risk, the obesity paradox should be re-evaluated using trajectories and changes in body weight and frailty levels as exposure or mediate variables, because reportedly the onset of frailty during the observation period may be a mediating mechanism between weight loss in midlife and increased mortality risk in old age [49].

We found that even individuals with frailty with optimal BMI showed a significantly shorter disability-free survival than did older individuals without frailty with a BMI of 21.5–24.9 kg/m^2^, suggesting that optimal BMI does not completely offset the risk of frailty-related adverse events. This suggested that the obesity paradox may not exist if it were to consider disability rather than mortality, as individuals with frailty and obesity have the longest duration of survival with disability. Maintaining the optimal BMI and improving frailty may contribute not only to prolonging life expectancy, but also shortening survival with disability in older adults. Frailty is a reversible condition wherein the individual can return to a healthy state through appropriate lifestyle intervention programmes [50]; therefore, mitigating frailty levels should be prioritised over attaining the optimal BMI in older individuals with frailty. Moreover, early detection of frailty using frailty screening tools by healthcare manager or professionals is necessary in clinical and public health settings. As the prevalence of subdomains related to frailty varies according to BMI [18], interventions should be personalized based on an individual’s BMI and frailty aspects.

## Conclusion

The BMI range with the lowest HR for disability was 22.5–23.5 kg/m^2^ in older adults with and without frailty, and higher and lower BMI was associated with disability. Older adults with BMI ≥ 27.5 kg/m^2^ had longer survival with disability, but those with BMI < 18.5 kg/m^2^ were more likely to die before disability incidence. This study suggests that reversal of frailty should be prioritised because even individuals with frailty with an optimal BMI face a higher risk of disability than do individuals without frailty with higher or lower BMI.

## Supplementary information

SUPPLEMENTAL MATERIAL

## Data Availability

The datasets described in the manuscript will be made available by the corresponding author (d2watanabe@nibiohn.go.jp), TY (t-yoshida@nibiohn.go.jp), and YY (yamaday@nibiohn.go.jp) on reasonable request.
