# Supplementary material for: Is a higher body mass index associated with longer duration of survival with disability in frail than in non-frail older adults?
Source: Int J Obes (Lond). 2024 Nov 15;49(2):348–56. doi: 10.1038/s41366-024-01681-6 (PMC11805705; doi:10.1038/s41366-024-01681-6)
Supplement: Supplementary file 1 — SUPPLEMENTAL MATERIAL [file 41366_2024_1681_MOESM1_ESM.docx]

Supporting information

Is a higher body mass index associated with longer duration of survival with disability in frail than in non-frail older adults?

Authors: Daiki Watanabe, Tsukasa Yoshida, Yuya Watanabe, Yosuke Yamada, and Misaka Kimura

**SUPPLEMENTARY TABLES AND FIGURES**

**Supplementary Table 1.** Prevalence rate of the subdomains the Kihon Checklist by body mass index.

**Supplementary Table 2.** Characteristics of the included and excluded participants.

**Supplementary Table 3.** Results of the sensitivity analysis for the relationship between body mass index and disability after excluding participants with an event in the first 2 years of follow-up.

**Supplementary Table 4.** Results of the sensitivity analysis for the relationship between body mass index and disability using a multiple imputation method for missing covariate values.

**Supplementary Table 5.** Sub-distribution hazard ratios for body mass index and disability, calculated using the multivariable Fine–Gray competing risk model.

**Supplementary Table 6.** The 50^th^ percentile differences in age at disability and death for frailty and body mass index status calculated using the multivariable Laplace regression model.

**Supplementary Figure 1.** Bland Altman plot for baseline body mass index calculated from the self-reported and measured height and body weight.

**Supplementary Table 1**. Prevalence rate of the subdomains the Kihon Checklist by body mass index.

|  | Total (*n* = 10232) | | BMI categories (kg/m^2^) | | | | | | | | | | p-value |
| --- | --- | --- | --- | --- | --- | --- | --- | --- | --- | --- | --- | --- | --- |
|  |  |  | <18.5 (*n* = 933) | | 18.5-21.4 (*n* = 2882) | | 21.5-24.9 (*n* = 4370) | | 25.0-27.4 (*n* = 1413) | | ≥ 27.5 (*n* = 634) | |  |
| IADL disability [n (%)] | 1413 | (13.8) | 286 | (30.7) | 427 | (14.8) | 440 | (10.1) | 150 | (10.6) | 110 | (17.4) | <0.001 |
| Physical [n (%)] | 2744 | (26.8) | 340 | (36.4) | 751 | (26.1) | 1021 | (23.4) | 379 | (26.8) | 253 | (39.9) | <0.001 |
| Nutrition [n (%)] | 249 | (2.4) | 249 | (26.7) | 0 | (0.0) | 0 | (0.0) | 0 | (0.0) | 0 | (0.0) | <0.001 |
| Oral [n (%)] | 2634 | (25.7) | 327 | (35.1) | 755 | (26.2) | 1033 | (23.6) | 334 | (23.6) | 185 | (29.2) | <0.001 |
| Social [n (%)] | 1008 | (9.9) | 181 | (19.4) | 323 | (11.2) | 317 | (7.3) | 107 | (7.6) | 80 | (12.6) | <0.001 |
| Cognitive [n (%)] | 3995 | (39.0) | 426 | (45.7) | 1149 | (39.9) | 1611 | (36.9) | 531 | (37.6) | 278 | (43.9) | <0.001 |
| Depression [n (%)] | 3502 | (34.2) | 433 | (46.4) | 1031 | (35.8) | 1331 | (30.5) | 437 | (30.9) | 270 | (42.6) | <0.001 |

IADL, Instrumental Activities of Daily Living; BMI, body mass index

All values are shown in terms of the number of cases with percentage and have been analysed using the Pearson's Chi-square test.

The cutoff points of the Kihon Checklist subdomains are the following: the cutoff point for IADL disability, physical inactivity, malnutrition, oral dysfunction, socialization domain, cognitive domain, and depression were defined as ≥10 points on 20 items, including shopping, ≥3 points on five items, including walk continuously and history of fall, 2 points on two items, including weight loss and low body mass index (<18.5 kg/m^2^), ≥2 points on three items, including dry mouth and poor mastication, ≥1 point on two items, including frequency of going out less, ≥1 point on three items, including memory loss, and ≥2 points on five items, including fulfilment and helplessness, respectively.

**Supplementary Table 2**. Characteristics of the included and excluded participants.

|  | All participants (*n* = 13294) | |  | Included participants (*n* = 10232) | | Excluded participants (*n* = 3062) | |
| --- | --- | --- | --- | --- | --- | --- | --- |
| Age [years] ^a^ | 74.5 | (6.9) |  | 73.6 | (6.5) | 77.7 | (7.4) |
| Women [*n* (%)] ^b^ | 7337 | (55.2) |  | 5459 | (53.4) | 1878 | (61.3) |
| PD ≥1000 people/km^2^ [*n* (%)] ^b^ | 5917 | (44.5) |  | 4616 | (45.1) | 1301 | (42.5) |
| Height [cm] ^a^ | 156.6 | (9.1) |  | 156.9 | (9.0) | 154.8 | (9.5) |
| Body weight [kg] ^a^ | 55.4 | (11.1) |  | 55.7 | (10.3) | 53.8 | (14.0) |
| Body mass index [kg/m^2^] ^a^ | 22.5 | (3.6) |  | 22.5 | (3.2) | 22.6 | (5.3) |
| Current smoker [*n* (%)] ^b^ | 1384 | (10.4) |  | 1169 | (11.4) | 215 | (8.4) |
| Current alcohol drinker [*n* (%)] ^b^ | 4675 | (35.2) |  | 3960 | (38.7) | 715 | (27.3) |
| Physical activity [min/week] ^a^ | 200 | (412) |  | 216 | (419) | 138 | (380) |
| Sitting time[min/day] ^a^ | 336 | (237) |  | 327 | (229) | 374 | (263) |
| Sleep time [min/day] ^a^ | 411 | (95) |  | 408 | (89) | 425 | (117) |
| Living alone [*n* (%)] ^b^ | 1643 | (12.4) |  | 1161 | (11.4) | 482 | (18.2) |
| Education ≥13 y [*n* (%)] ^b^ | 2448 | (18.4) |  | 2076 | (20.3) | 372 | (16.9) |
| HSES [*n* (%)] ^b^ | 4136 | (31.1) |  | 3254 | (31.8) | 882 | (32.7) |
| Denture use [*n* (%)] ^b^ | 8058 | (60.6) |  | 6095 | (59.6) | 1963 | (70.9) |
| No medication [*n* (%)] ^b^ | 2545 | (19.1) |  | 2134 | (20.9) | 411 | (16.6) |
| Hypertension [*n* (%)] ^b^ | 4903 | (36.9) |  | 3920 | (38.3) | 983 | (32.1) |
| Stroke [*n* (%)] ^b^ | 617 | (4.6) |  | 429 | (4.2) | 188 | (6.1) |
| Heart disease [*n* (%)] ^b^ | 1659 | (12.5) |  | 1261 | (12.3) | 398 | (13.0) |
| Diabetes [*n* (%)] ^b^ | 1390 | (10.5) |  | 1088 | (10.6) | 302 | (9.9) |
| Hyperlipidaemia [*n* (%)] ^b^ | 1122 | (8.4) |  | 970 | (9.5) | 152 | (5.0) |
| Respiratory disease [*n* (%)] ^b^ | 667 | (5.0) |  | 482 | (4.7) | 185 | (6.0) |
| Digestive disease [*n* (%)] ^b^ | 1043 | (7.8) |  | 850 | (8.3) | 193 | (6.3) |
| Urological diseases [*n* (%)] ^b^ | 817 | (6.1) |  | 640 | (6.3) | 177 | (5.8) |
| Cancer [*n* (%)] ^b^ | 487 | (3.7) |  | 387 | (3.8) | 100 | (3.3) |
| No. of chronic diseases ^a,c^ | 0.96 | (1.00) |  | 0.98 | (0.99) | 0.87 | (1.00) |

PD, population density; HSES, high socioeconomic status

The number of missing values for variables are as follows [*n* = (*n* in included participants) and (*n* in excluded participants)]: height (*n* = 0 and 935); body weight (*n* = 0 and 821); body mass index (*n* = 0 and 1039); smoking status (*n* = 195 and 507); alcohol drinker (*n* = 161 and 443); physical activity (*n* = 228 and 326); sitting time (*n* = 1,075 and 820); sleep time (*n* = 484 and 553); family structure (*n* = 709 and 410); educational attainment (*n* = 1,034 and 861); socioeconomic status (*n* = 369 and 361); denture use (*n* = 111 and 294); and medications (*n* = 558 and 582). Body mass index was calculated as body weight (kg) divided by height squared (m^2^).

^a^ Continuous values are presented as the mean (standard deviation).

^b^ Categorical values are presented as numbers (percentages).

^c^ The comorbidity scores were summed to obtain a total score ranging from 0 (no comorbidity) to 9 (poor status) from the data obtained on disease status (including the presence of hypertension, stroke, heart disease, diabetes, hyperlipidaemia, digestive disease, respiratory disease, urological diseases, and cancer).

**Supplementary Table 3**. Results of the sensitivity analysis for the relationship between body mass index and disability after excluding participants with an event in the first 2 years of follow-up.

| **BMI group** | *n* | Event | PY | Event/1000 PY | | Model 1^a^ | | Model 2^b^ | |
| --- | --- | --- | --- | --- | --- | --- | --- | --- | --- |
|  |  |  |  | Rate | 95%CI | HR | 95%CI | HR | 95%CI |
| ***Total*** |  |  |  |  |  |  |  |  |  |
| < 18.5 | 734 | 163 | 3352 | 48.6 | (41.7 to 56.7) | 1.47 | (1.23 to 1.75) | 1.29 | (1.08 to 1.55) |
| 18.5-21.4 | 2538 | 331 | 12312 | 26.9 | (24.1 to 29.9) | 0.94 | (0.82 to 1.08) | 0.95 | (0.82 to 1.09) |
| 21.5-24.9 | 3994 | 493 | 19839 | 24.9 | (22.8 to 27.1) | 1.00 | (Ref) | 1.00 | (Ref) |
| 25.0-27.4 | 1292 | 144 | 6378 | 22.6 | (19.2 to 26.6) | 0.89 | (0.74 to 1.07) | 0.85 | (0.71 to 1.03) |
| ≥ 27.5 | 556 | 99 | 2682 | 36.9 | (30.3 to 45.0) | 1.59 | (1.28 to 1.97) | 1.36 | (1.10 to 1.70) |
| *p for trend*^c^ |  |  |  |  |  | 0.949 | | 0.625 | |
| *p for non-linearity* |  |  |  |  |  | <0.001 | | 0.001 | |
| ***Frailty***^d^ |  |  |  |  |  |  |  |  |  |
| < 18.5 | 393 | 122 | 1668 | 73.1 | (61.2 to 87.3) | 1.19 | (0.97 to 1.48) | 1.22 | (0.98 to 1.51) |
| 18.5-21.4 | 882 | 199 | 3962 | 50.2 | (43.7 to 57.7) | 0.86 | (0.72 to 1.03) | 0.89 | (0.74 to 1.06) |
| 21.5-24.9 | 1216 | 291 | 5650 | 51.5 | (45.9 to 57.8) | 1.00 | (Ref) | 1.00 | (Ref) |
| 25.0-27.4 | 428 | 90 | 2011 | 44.8 | (36.4 to 55.0) | 0.87 | (0.69 to 1.10) | 0.86 | (0.67 to 1.08) |
| ≥ 27.5 | 251 | 66 | 1168 | 56.5 | (44.4 to 71.9) | 1.24 | (0.95 to 1.61) | 1.19 | (0.90 to 1.56) |
| *p for trend*^c^ |  |  |  |  |  | 0.958 | | 0.598 | |
| *p for non-linearity* |  |  |  |  |  | 0.004 | | 0.008 | |
| ***Non-frailty***^d^ |  |  |  |  |  |  |  |  |  |
| < 18.5 | 341 | 41 | 1684 | 24.3 | (17.9 to 33.1) | 1.51 | (1.07 to 2.11) | 1.48 | (1.05 to 2.08) |
| 18.5-21.4 | 1656 | 132 | 8350 | 15.8 | (13.3 to 18.7) | 1.04 | (0.84 to 1.30) | 1.06 | (0.84 to 1.32) |
| 21.5-24.9 | 2778 | 202 | 14188 | 14.2 | (12.4 to 16.3) | 1.00 | (Ref) | 1.00 | (Ref) |
| 25.0-27.4 | 864 | 54 | 4366 | 12.4 | (9.5 to 16.1) | 0.86 | (0.64 to 1.16) | 0.83 | (0.61 to 1.12) |
| ≥ 27.5 | 305 | 33 | 1514 | 21.8 | (15.5 to 30.7) | 1.79 | (1.24 to 2.59) | 1.73 | (1.19 to 2.51) |
| *p for trend*^c^ |  |  |  |  |  | 0.915 | | 0.661 | |
| *p for non-linearity* |  |  |  |  |  | 0.001 | | 0.003 | |

BMI, body mass index; PY, person-years; CI, confidence interval; HR, hazard ratio; Ref, reference

^a^ Model 1: Adjusted for age, sex, and population density.

^b^ Model 2: Adjusted for the variables of Model 1 and smoking status, alcohol consumption status, physical activity, sitting time, sleep time, family structure, educational attainment, economic status, denture use, medication use, number of chronic diseases, and/or frailty status.

^c^ Linear trend p-values were calculated using the likelihood ratio test and BMI as a continuous variable.

^d^ Variables (frailty) used for subgroup analysis were excluded from the adjustment for covariate variables in the model.

**Supplementary Table 4.** Results of the sensitivity analysis for the relationship between body mass index and disability using a multiple imputation method for missing covariate values.

| **BMI group** | *n* | Event | PY | Event/1000 PY | | Model 1^a^ | | Model 2^b^ | |
| --- | --- | --- | --- | --- | --- | --- | --- | --- | --- |
|  |  |  |  | Rate | 95%CI | HR | 95%CI | HR | 95%CI |
| ***Total*** |  |  |  |  |  |  |  |  |  |
| < 18.5 | 933 | 362 | 3497 | 103.5 | (93.4 to 114.7) | 1.50 | (1.32 to 1.70) | 1.31 | (1.16 to 1.49) |
| 18.5-21.4 | 2882 | 675 | 12604 | 53.6 | (49.7 to 57.8) | 1.00 | (0.90 to 1.10) | 0.99 | (0.90 to 1.10) |
| 21.5-24.9 | 4370 | 869 | 20152 | 43.1 | (40.3 to 46.1) | 1.00 | (Ref) | 1.00 | (Ref) |
| 25.0-27.4 | 1413 | 265 | 6482 | 40.9 | (36.2 to 46.1) | 0.93 | (0.81 to 1.07) | 0.88 | (0.77 to 1.01) |
| ≥ 27.5 | 634 | 177 | 2736 | 64.7 | (55.8 to 74.9) | 1.56 | (1.33 to 1.84) | 1.28 | (1.08 to 1.50) |
| *p for trend* ^c^ | |  |  |  |  | 0.239 | | 0.066 | |
| *p for non-linearity* | |  |  |  |  | <0.001 | | <0.001 | |
| ***Frailty***^d^ |  |  |  |  |  |  |  |  |  |
| < 18.5 | 575 | 304 | 1790 | 169.9 | (151.8 to 190.1) | 1.23 | (1.07 to 1.42) | 1.28 | (1.11 to 1.47) |
| 18.5-21.4 | 1170 | 487 | 4189 | 116.3 | (106.4 to 127.1) | 0.95 | (0.84 to 1.07) | 0.98 | (0.86 to 1.10) |
| 21.5-24.9 | 1516 | 591 | 5888 | 100.4 | (92.6 to 108.8) | 1.00 | (Ref) | 1.00 | (Ref) |
| 25.0-27.4 | 521 | 183 | 2081 | 87.9 | (76.1 to 101.6) | 0.89 | (0.76 to 1.06) | 0.87 | (0.74 to 1.03) |
| ≥ 27.5 | 323 | 138 | 1216 | 113.5 | (96.0 to 134.1) | 1.28 | (1.06 to 1.54) | 1.21 | (1.00 to 1.46) |
| *p for trend* ^c^ | |  |  |  |  | 0.493 | | 0.083 | |
| *p for non-linearity* | |  |  |  |  | <0.001 | | <0.001 | |
| ***Non-frailty***^d^ |  |  |  |  |  |  |  |  |  |
| < 18.5 | 358 | 58 | 1708 | 34.0 | (26.3 to 43.9) | 1.48 | (1.11 to 1.98) | 1.46 | (1.09 to 1.95) |
| 18.5-21.4 | 1712 | 188 | 8415 | 22.3 | (19.4 to 25.8) | 1.06 | (0.88 to 1.28) | 1.07 | (0.89 to 1.29) |
| 21.5-24.9 | 2854 | 278 | 14264 | 19.5 | (17.3 to 21.9) | 1.00 | (Ref) | 1.00 | (Ref) |
| 25.0-27.4 | 892 | 82 | 4401 | 18.6 | (15.0 to 23.1) | 0.94 | (0.74 to 1.21) | 0.92 | (0.71 to 1.17) |
| ≥ 27.5 | 311 | 39 | 1520 | 25.7 | (18.7 to 35.1) | 1.52 | (1.08 to 2.12) | 1.42 | (1.01 to 1.99) |
| *p for trend* ^c^ | |  |  |  |  | 0.511 | | 0.257 | |
| *p for non-linearity* | |  |  |  |  | <0.001 | | 0.001 | |

BMI, body mass index; PY, person-years; CI, confidence interval; HR, hazard ratio; Ref, reference

^a^ Model 1: Adjusted for age, sex, and population density.

^b^ Model 2: Adjusted for the variables from Model 1 and smoking status, alcohol consumption status, physical activity, sitting time, sleep time, family structure, educational attainment, economic status, denture use, medication use, number of chronic diseases, and/or frailty status.

^c^ Linear trend p-values were calculated using the likelihood ratio test and BMI as a continuous variable.

^d^ Variables (frailty) used for subgroup analysis were excluded from the adjustment for covariate variables in the model.

**Supplementary Table 5**. Sub-distribution hazard ratios for body mass index and disability, calculated using the multivariable Fine–Gray competing risk model.

| **BMI group** | *n* | Event | PY | Event/1000 PY | | Model 1^a^ | | Model 2^b^ | |
| --- | --- | --- | --- | --- | --- | --- | --- | --- | --- |
|  |  |  |  | Rate | 95%CI | SHR | 95%CI | SHR | 95%CI |
| ***Total*** |  |  |  |  |  |  |  |  |  |
| < 18.5 | 933 | 362 | 3497 | 103.5 | (93.4 to 114.7) | 1.42 | (1.25 to 1.62) | 1.25 | (1.09 to 1.43) |
| 18.5-21.4 | 2882 | 675 | 12604 | 53.6 | (49.7 to 57.8) | 0.96 | (0.87 to 1.07) | 0.96 | (0.87 to 1.07) |
| 21.5-24.9 | 4370 | 869 | 20152 | 43.1 | (40.3 to 46.1) | 1.00 | (Ref) | 1.00 | (Ref) |
| 25.0-27.4 | 1413 | 265 | 6482 | 40.9 | (36.2 to 46.1) | 0.93 | (0.81 to 1.07) | 0.89 | (0.77 to 1.02) |
| ≥ 27.5 | 634 | 177 | 2736 | 64.7 | (55.8 to 74.9) | 1.58 | (1.33 to 1.87) | 1.30 | (1.10 to 1.54) |
| *p for trend^c^* |  |  |  |  |  | 0.823 | | 0.456 | |
| *p for non-linearity* |  |  |  |  |  | <0.001 | | <0.001 | |
| ***Frailty***^d^ |  |  |  |  |  |  |  |  |  |
| < 18.5 | 575 | 304 | 1790 | 169.9 | (151.8 to 190.1) | 1.17 | (1.02 to 1.35) | 1.21 | (1.04 to 1.40) |
| 18.5-21.4 | 1170 | 487 | 4189 | 116.3 | (106.4 to 127.1) | 0.91 | (0.81 to 1.03) | 0.95 | (0.83 to 1.07) |
| 21.5-24.9 | 1516 | 591 | 5888 | 100.4 | (92.6 to 108.8) | 1.00 | (Ref) | 1.00 | (Ref) |
| 25.0-27.4 | 521 | 183 | 2081 | 87.9 | (76.1 to 101.6) | 0.90 | (0.76 to 1.07) | 0.88 | (0.74 to 1.04) |
| ≥ 27.5 | 323 | 138 | 1216 | 113.5 | (96.0 to 134.1) | 1.29 | (1.07 to 1.56) | 1.23 | (1.02 to 1.49) |
| *p for trend^c^* |  |  |  |  |  | 0.814 | | 0.067 | |
| *p for non-linearity* |  |  |  |  |  | <0.001 | | <0.001 | |
| ***Non-frailty***^d^ |  |  |  |  |  |  |  |  |  |
| < 18.5 | 358 | 58 | 1708 | 34.0 | (26.3 to 43.9) | 1.46 | (1.10 to 1.93) | 1.45 | (1.09 to 1.94) |
| 18.5-21.4 | 1712 | 188 | 8415 | 22.3 | (19.4 to 25.8) | 1.04 | (0.86 to 1.25) | 1.05 | (0.87 to 1.26) |
| 21.5-24.9 | 2854 | 278 | 14264 | 19.5 | (17.3 to 21.9) | 1.00 | (Ref) | 1.00 | (Ref) |
| 25.0-27.4 | 892 | 82 | 4401 | 18.6 | (15.0 to 23.1) | 0.93 | (0.73 to 1.20) | 0.90 | (0.70 to 1.16) |
| ≥ 27.5 | 311 | 39 | 1520 | 25.7 | (18.7 to 35.1) | 1.51 | (1.08 to 2.12) | 1.43 | (1.02 to 2.01) |
| *p for trend^c^* |  |  |  |  |  | 0.664 | | 0.364 | |
| *p for non-linearity* |  |  |  |  |  | <0.001 | | 0.003 | |

BMI, body mass index; CI, confidence interval; PY, person-years; Ref, reference; SHR, sub-distribution hazard ratio

^a^ Model 1: Adjusted for age, sex, and population density.

^b^ Model 2: Adjusted for the variables from Model 1 and smoking status, alcohol consumption status, physical activity, sitting time, sleep time, family structure, educational attainment, economic status, denture use, medication use, number of chronic diseases, and/or frailty status.

^c^ Linear trend p-values were calculated using the likelihood ratio test and BMI as a continuous variable.

^d^ Variables (frailty) used for subgroup analysis were excluded from the adjustment for covariate variables in the model.

**Supplementary Table 6.** The 50^th^ percentile differences in age at disability and death for frailty and BMI status calculated using the multivariable Laplace regression model.

| **BMI  group** | *n* | Mortality | | | | Disability | | | | Survival with disability ^b^ |
| --- | --- | --- | --- | --- | --- | --- | --- | --- | --- | --- |
|  |  | Event/1000 PY | | Overall survival (months)^a^ | | Event/1000 PY | | Disability-free survival (months)^a^ | |  |
|  |  | Rate | 95%CI | 50^th^ PD (95%CI) | | Rate | 95%CI | 50^th^ PD (95%CI) | |  |
| ***Total*** |  |  |  |  |  |  |  |  |  |  |
| < 18.5 | 933 | 48.3 | (42.2 to 55.3) | -17.3 | (-22.1 to -12.4) | 103.5 | (93.4 to 114.7) | -7.1 | (-11.0 to -3.2) | -10.2 |
| 18.5-21.4 | 2882 | 25.4 | (22.9 to 28.2) | -7.6 | (-11.4 to -3.8) | 53.6 | (49.7 to 57.8) | 0.4 | (-2.7 to 3.6) | -8.0 |
| 21.5-24.9 | 4370 | 16.2 | (14.7 to 18.0) | 0.0 | (Ref) | 43.1 | (40.3 to 46.1) | 0.0 | (Ref) | Ref |
| 25.0-27.4 | 1413 | 13.8 | (11.3 to 16.8) | 4.1 | (-1.2 to 9.5) | 40.9 | (36.2 to 46.1) | 2.8 | (-1.5 to 7.1) | 1.3 |
| ≥ 27.5 | 634 | 15.5 | (11.7 to 20.4) | 3.5 | (-3.7 to 10.8) | 64.7 | (55.8 to 74.9) | -9.0 | (-14.5 to -3.4) | 12.5 |
| **Frailty status**^c^ | |  |  |  |  |  |  |  |  |  |
| ***Frailty*** |  |  |  |  |  |  |  |  |  |  |
| < 18.5 | 575 | 72.6 | (62.8 to 83.9) | -32.9 | (-39.6 to -26.2) | 169.9 | (151.8 to 190.1) | -33.4 | (-39.2 to -27.6) | 0.5 |
| 18.5-21.4 | 1170 | 46.3 | (40.9 to 52.3) | -25.0 | (-31.1 to -18.8) | 116.3 | (106.4 to 127.1) | -28.7 | (-33.9 to -23.5) | 3.7 |
| 21.5-24.9 | 1516 | 30.1 | (26.4 to 34.4) | -13.9 | (-19.5 to -8.3) | 100.4 | (92.6 to 108.8) | -28.0 | (-32.8 to -23.3) | 14.1 |
| 25.0-27.4 | 521 | 20.4 | (15.6 to 26.7) | -9.8 | (-19.1 to -0.5) | 87.92 | (76.1 to 101.6) | -25.4 | (-30.9 to -19.9) | 15.6 |
| ≥ 27.5 | 323 | 20.2 | (14.4 to 28.4) | -7.3 | (-17.6 to 3.0) | 113.5 | (96.0 to 134.1) | -34.5 | (-41.6 to -27.5) | 27.2 |
| ***Non-frailty*** | |  |  |  |  |  |  |  |  |  |
| < 18.5 | 358 | 14.3 | (9.8 to 21.0) | -9.9 | (-19.5 to -0.2) | 33.96 | (26.3 to 43.9) | -11.1 | (-19.7 to -2.6) | 1.2 |
| 18.5-21.4 | 1712 | 12.4 | (10.3 to 14.9) | -7.9 | (-14.0 to -1.8) | 22.34 | (19.4 to 25.8) | -3.8 | (-9.1 to 1.4) | -4.1 |
| 21.5-24.9 | 2854 | 9.3 | (7.8 to 11.0) | 0.0 | (Ref) | 19.49 | (17.3 to 21.9) | 0.0 | (Ref) | Ref |
| 25.0-27.4 | 892 | 10.0 | (7.5 to 13.4) | -0.4 | (-9.0 to 8.1) | 18.63 | (15.0 to 23.1) | 3.4 | (-3.1 to 9.9) | -3.8 |
| ≥ 27.5 | 311 | 10.7 | (6.6 to 17.1) | -3.0 | (-15.5 to 9.5) | 25.66 | (18.7 to 35.1) | -9.2 | (-19.1 to 0.8) | 6.2 |

BMI, body mass index; CI, confidence interval; PD, percentile difference; PY, person-years; Ref, reference

^a^ Model 2: Adjusted for age, sex, population density, smoking status, alcohol consumption status, physical activity, sitting time, sleep time, family structure, educational attainment, economic status, denture use, medication use, number of chronic diseases, and/or frailty status.

^b^ To calculate the duration of survival with disability, we calculated the difference in the 50^th^ PD of disability and death events using the following equation: 50^th^ PD of overall survival − 50^th^ PD of disability-free survival. If the results are greater than 0 (value is +), the duration of survival with disability is inferred to be longer. If the value is lower than 0, the participant is more likely to die before disability incidence.

^c^ Variables (frailty) used for subgroup analysis were excluded from the adjustment for covariate variables in the model. These results are presented as PD (95% CI), with the PD calculated using a non-frail individual with BMI=21.5–24.9 kg/m^2^ as the reference.


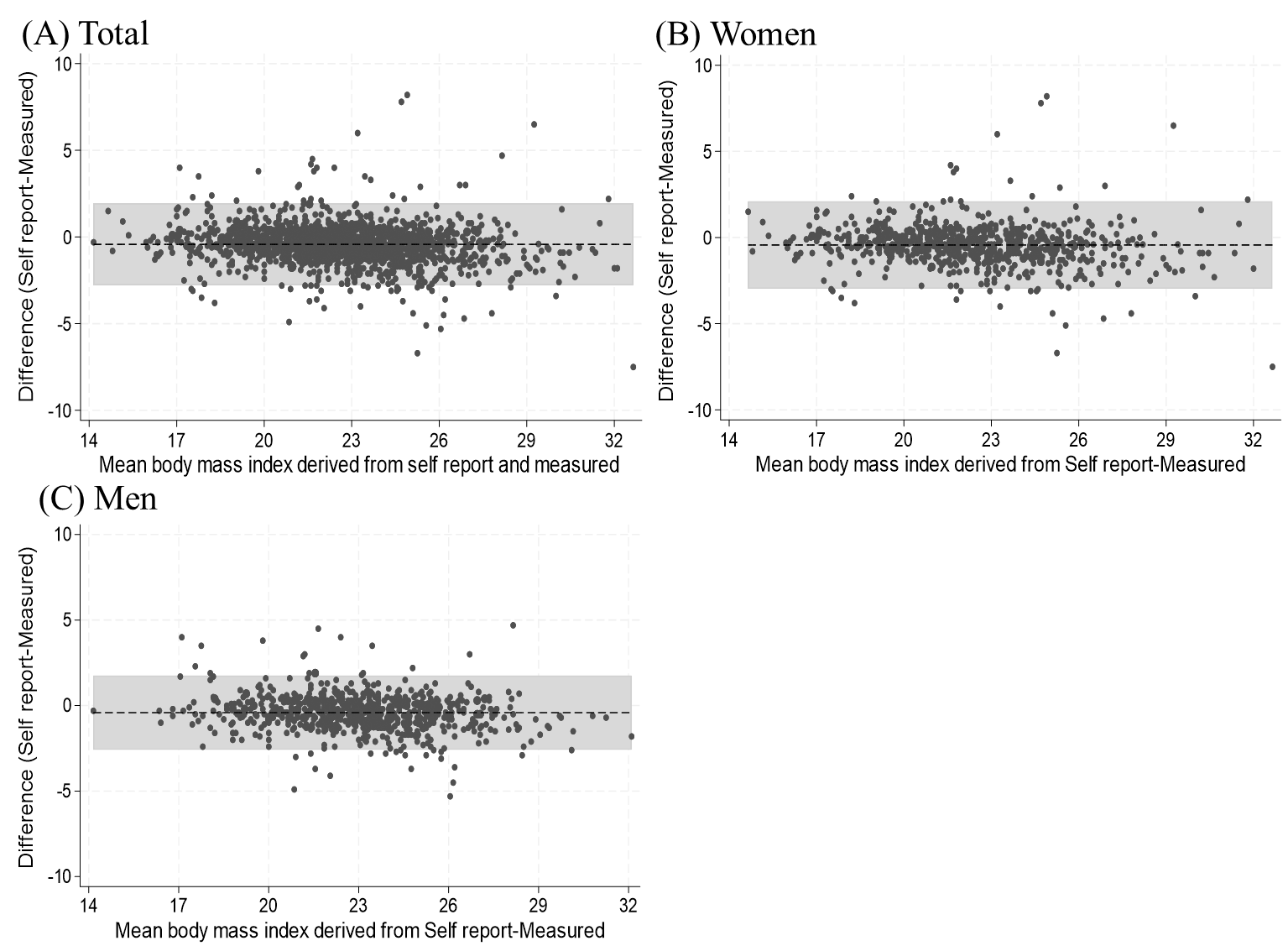


**Supplementary Figure 1**. Bland Altman plot for baseline body mass index calculated from the self-reported and measured heigh and body weight.

The centred dotted line represents the mean differences and the grey shades represent the upper and lower 95% of limits of agreement. (A) Total (n = 1368); Mean difference: -0.4, limits of agreement: -2.8 to 1.9, (B) Women (n = 694); Mean difference: -0.4, limits of agreement: -3.0 to 2.1, and Men (n = 674); Mean difference: -0.4, limits of agreement: -2.6 to 1.8.
